# The highest percentage of Gleason Pattern 4 is a predictor in intermediate‐risk prostate cancer

**DOI:** 10.1002/bco2.195

**Published:** 2022-10-17

**Authors:** Shun Sato, Takahiro Kimura, Hajime Onuma, Shin Egawa, Masayuki Shimoda, Hiroyuki Takahashi

**Affiliations:** ^1^ Department of Pathology The Jikei University School of Medicine Tokyo Japan; ^2^ Department of Urology The Jikei University School of Medicine Tokyo Japan

**Keywords:** cribriform pattern, Gleason score, pathological marker, percentage of Gleason Pattern 4, prognostic factor, prostate cancer

## Abstract

**Objectives:**

This study aims to clarify the clinicopathological significance of several novel pathological markers, including the percentage of Gleason pattern 4 and small/non‐small cribriform pattern, in intermediate‐risk Gleason score 3 + 4 = 7 prostate cancer.

**Subjects and Methods:**

Two‐hundred and twenty‐eight patients with Gleason score 3 + 4 = 7 intermediate‐risk prostate cancer who underwent radical prostatectomy between 2009 and 2019 at our institute were selected. Preoperative clinicopathological characteristics, including serum prostate‐specific antigen level, clinical T stage, percentage of cancer‐positive cores at biopsy, small/non‐small cribriform pattern, the highest percentage of Gleason pattern 4, the total length of Gleason pattern 4 and percentage of Gleason score 7 cores were examined in univariate/multivariate logistic regression analysis to determine their predictive value for postoperative adverse pathological findings, defined as an upgrade to Gleason score 4 + 3 = 7 or higher, pN1 or pT3b disease.

**Results:**

Fifty‐four cases (23.7%) showed adverse pathological findings. Although a non‐small cribriform pattern, highest Gleason pattern 4 percentage and total length of Gleason pattern 4 were predictive of adverse pathological findings in univariate analysis, only the highest Gleason pattern 4 percentage was an independent predictive factor in multivariate analysis (odds ratio: 1.610; 95% confidence interval: 1.260–2.070; *P* = 0.0002).

**Conclusion:**

The highest Gleason pattern 4 percentage was a potent predictive parameter for Gleason score 3 + 4 = 7 intermediate‐risk prostate cancer and should be considered in the risk classification scheme for prostate cancer.

## 1 INTRODUCTION

In prostate cancer management, treatment options are selected according to risk classifications, which are defined by several clinicopathological factors.^1^, ^2^, ^3^ The earliest D'Amico classification system, introduced in many countries and used for many lesions, adopts a three‐tier classification, dividing prostate cancers into low‐, intermediate‐ and high‐risk categories.^1^ However, intermediate‐risk prostate cancer (IR‐PCa), including cancers with a Gleason score (GS) of 3 + 4 = 7, has been shown to encompass a variety of cases with different biological behaviours. Thus, recent deliberations have attempted to address the subclassification of IR‐PCa, which will provide clinicians with a novel decision‐making strategy that includes the application of active surveillance for more indolent cancers.^4^ For example, the latest National Comprehensive Cancer Network (NCCN) guideline, which is the most widely distributed worldwide, divides IR‐PCa into two favourable and unfavourable categories defined by clinical T stage (cT), serum prostate‐specific antigen (PSA) level, GS at biopsy and percentage of positive cores. Favourable intermediate‐risk disease is defined as follows: IR‐PCa with GS ≤ 3 + 4 = 7, percentage of positive cores <50% and only one intermediate‐risk feature of cT2b‐2c, GS 7 and PSA 10–20 ng/dl. In the NCCN guideline, different treatment strategies are assigned for favourable IR‐PCa. For example, one option is active surveillance.^2^

Recent studies have shown that an increased percentage of Gleason Pattern 4 (%GP4) and the presence of a cribriform pattern (CP) in biopsy specimens are associated with a worse prognosis and a high prevalence of adverse pathological (AP) findings in radical prostatectomy specimens in GS 7 cancers.^5^, ^6^, ^7^, ^8^, ^9^, ^10^, ^11^, ^12^ These pathological parameters play a significant role in the treatment decision‐making process, especially in GS 3 + 4 = 7 IR‐PCa. In fact, some guidelines from North America adopt %GP4 as an inclusion criterion for active surveillance in GS 3 + 4 = 7 patients.^13^, ^14^ However, few studies have examined the clinicopathological significance of these pathological factors, including %GP4 and CP, in IR‐PCa. Therefore, these factors' clinical impact and predictive value in GS 3 + 4 = 7 IR‐PCa have not been validated.

In terms of pathological diagnosis, cribriform glands of similar or smaller size to non‐neoplastic acini were newly assigned Gleason Pattern 4 instead of the previously assigned Gleason Pattern 3 in the 2014 International Society of Urological Pathology Consensus (ISUP 2014), which has been the gold standard for current pathological diagnosis of prostate cancer.^15^ However, the evidence to justify this change is very limited. Regarding the clinicopathological significance of the size of CP, Iczkowski et al. reported that small CP, which was defined as CP with both 12 or less lumens and no solid lesions, in radical prostatectomy specimens was associated with a higher risk of biochemical recurrence.^16^ However, their cohort included high‐risk/higher‐grade cases with GS ≥ 4 + 3 = 7. In addition, all cases of the small CP category in Iczkowski et al.'s study also included non‐small CP. Thus, the actual clinicopathological significance of small CP in IR‐PCa has not yet been determined.

In this study, we comprehensively analysed clinicopathological parameters, including %GP4 and CP size, to identify the parameters with predictive significance for GS 3 + 4 = 7 IR‐PCa.

## 2 SUBJECTS AND METHODS

### 2.1 Patients

A total of 228 IR‐PCa patients with GS 3 + 4 = 7 at biopsy who underwent radical prostatectomy from 2009 to 2019 at our institute were included. Patients with any history of neoadjuvant endocrine therapy, chemotherapy or radiation therapy were excluded. In our institute, patients with suspicious anterior located tumours by magnetic resonance imaging (MRI) examination and those with any history of negative biopsy underwent transperineal saturation biopsy instead of routine transrectal sextant biopsy. Biopsy and radical prostatectomy specimens were available for pathological review in all the cases. Cases were specifically selected from the institutional medical records as follows: First, all biopsy specimens of 475 prostate cancer patients with a GS of 6 or 7 at biopsy were reviewed, and GS was assigned according to ISUP 2014.^15^ After the review, 242 patients with GS 3 + 4 = 7 were selected, and their medical records were checked for NCCN classification. Eventually, 14 high‐risk cases were excluded, and 228 patients were included in this study.

### 2.2 Acquisition of clinical information

For each patient, data pertaining to age, serum PSA level, cT and biopsy method were collected from their medical records. For those who underwent MRI examination before surgery, the cT stage was decided by integrating the findings of MRI, digital rectal examination and ultrasound.

### 2.3 Pathological assessment

**FIGURE 1:**
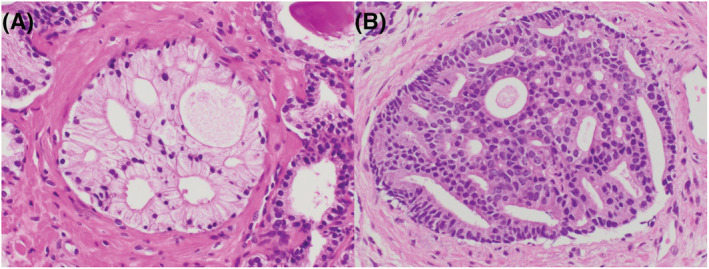
(A) The small cribriform gland pattern, which consisted of 12 or less lumens and did not include a solid component. (B) The non‐small cribriform gland, which consisted of many lumens

The haematoxylin and eosin (HE)‐stained sections of radical prostatectomy specimens were prepared according to the routine procedure at our institute, as reported previously.^5^ Biopsy and radical prostatectomy specimens were reviewed, and GS was assigned according to ISUP 2014 for all cases.^15^ In the biopsy specimens, tumour length, GS, %GP4 and the presence of small/non‐small CP were evaluated for all cancer‐positive cores. GS and %GP4 were assigned based on the agreement of two genitourinary pathologists (S.S. and H.T.). A small CP was defined as a ‘cribriform pattern with 12 or less lumens without solid lesion’ (Figure 1A), as previously reported.^16^, ^17^ All CPs other than small CPs were categorised as non‐small‐CPs (Figure 1B). Intraductal carcinoma was included in patients with non‐small CPs.

### 2.4 Outcome measures

AP findings in radical prostatectomy specimens were adopted as the outcome measure in this study. AP was defined as GS 4 + 3 = 7 or higher, pT3b staging or positive lymph node metastasis because these factors have been reported to be strongly associated with worse outcomes.^18^, ^19^

### 2.5 Statistical analysis and definition of clinicopathological parameters

Preoperative serum PSA level, cT, percentage of positive cores at biopsy, highest %GP4, percentage of GS 7 cores among all cores, the total length of GP4 (GP4‐TL) and presence of small/non‐small CP were compared in univariate and multivariate logistic regression analysis to determine their predictive value for AP. In the analysis, serum PSA levels and cT were divided into two categories: PSA < 10 and 10–20 ng/dl; cT1c/2a and cT2b/2c according to the NCCN risk classification. In multivariate analysis, a backward stepwise selection method was used. Spearman's rank correlation coefficient was calculated among the three different GP4 quantification methods, such as highest %GP4, percentage of GS 7 cores among all cores, and GP4‐TL, to examine the correlations between these methods. The highest %GP4 was defined as the largest percentage of GP4 among all the cores in each case. The GP4‐TL was calculated as follows: sum of tumour length × %GP4 in each core. Specific cut‐off values for continuous variables shown to be independent predictive factors for AP were calculated using the receiver operating characteristic (ROC) curve. In all analyses, statistical significance was defined as a *P*‐value < 0.05. The EZR software package based on R (R Foundation for Statistical Computing, Vienna, Austria) was used for analyses.^20^

### 2.6 Research ethics

This study was approved by the ethics committee of the affiliated institution (institutional ID number: 30‐043), which waived the need to obtain informed consent because of the retrospective study design and the absence of patient‐identifying information.

## 3 RESULTS

### 3.1 Patient background

Data for preoperative clinical characteristics, pathological findings on biopsy and radical prostatectomy specimens are shown in Tables 1 and 2 and Table S1. The median serum PSA level was 7.335 ng/ml, and 168 patients (73.8%) showed a PSA level < 10 ng/dl. Regarding cT, 202 patients (88.6%) had cT1c/2a disease. Accordingly, 125 cases (54.8%) showed NCCN favourable IR‐PC. For 37 patients (16.2%), any method of target biopsy, including MRI‐ultrasound fusion biopsy for only one case, was added to conventional systemic biopsy. Histological examination of the biopsy specimens revealed CP in 42 cases (18.4%), of which 26 cases (11.4%) included only small CPs. In the radical prostatectomy specimens, AP was observed in 54 cases (23.7%), of which 14 cases (6.1%) showed pT3b disease, two cases (0.9%) showed lymph node metastasis and 44 cases (19.3%) showed an upgrade to GS 4 + 3 = 7 or higher. The pT3b/N1 cases decreased from our previous cohort due to the exclusion of high‐risk cases, despite the increase in cohort size.^5^ By the NCCN risk features, AP was observed in 28 cases (22.4%) of favourable IR‐PC and 26 cases (25.2%) of unfavourable IR‐PC.

**TABLE 1:** Preoperative characteristics

Age^a^ (median)	60.75–69 (65)	Biopsy method	
NCCN favourable IR‐PC	125 cases (54.8%)	Transrectal	162 cases (71.1%)
PSA^a^ (median)	5.443–10.07 (7.335) ng/dl	Transperineal	66 cases (28.9%)
<10 ng/dl	168 cases (73.7%)	Number of cores retrieved^a^ (median)	12–22 (12)
10–20 ng/dl	60 cases (26.3%)	% positive cores^a^ (median)	14.1–41.7 (25.0)
Clinical T stage		Any cribriform pattern	42 cases (18.4%)
cT1c/2a	202 cases (88.6%)	Small cribriform pattern only	26 cases (11.4%)
cT2b/2c	26 cases (11.4%)	Non‐small cribriform pattern/IDC	16 cases (7.0%)
		Total length of GP4^a^	0.2–1.41 (0.575) mm
		% GS 7 cores^a^	8.3–17.0 (9.1)

^a^ Continuous variables are shown in interquartile range associated with median value in the parenthesis.

**TABLE 2:** Pathological characteristics in the radical prostatectomy specimen

Adverse pathology	54 cases (23.7%)	Gleason score	
Pathological T stage		GS 6	19 cases (8.3%)
pT2	134 cases (58.8%)	GS 3 + 4 = 7	165 cases (72.4%)
pT3a	80 cases (35.1%)	GS 4 + 3 = 7	33 case (14.5%)
pT3b	14 cases (6.1%)	GS 8	3 cases (1.3%)
Lymph nodes metastasis		≥ GS 9	8 cases (3.5%)
pN0	185 cases (81.1%)		
pN1	2 cases (0.9%)		
pNX	41 cases (18.0%)		

### 3.2 Predictive impact of each clinicopathological parameter on postoperative AP findings

The results of the univariate and multivariate logistic regression analyses of the predictive value for AP are shown in Table 3. In the univariate analysis, the presence of non‐small CP, the highest %GP4 and GP4‐TL were predictive of AP. Small CP was not predictive of AP even in the univariate analysis. Among them, only the highest %GP4 was an independent predictive factor in multivariate analysis (*P* = 0.0002). Serum PSA level, cT, percentage of positive cores and percentage of GS 7 cores were not predictive of AP in both univariate and multivariate analyses.

**TABLE 3:** Results of univariate and multivariate logistic regression analysis for the risk of adverse pathology

Clinicopathological parameters	Univariate analysis		Multivariate	
	Odds ratio (95% CI)	P‐value	Odds ratio (95% CI)	P‐value
PSA (10–20 ng/dl vs. <10 ng/dl)	1.400 (0.716–2.730)	0.325		
cT stage (2b + 2c vs. 1c + 2a)	0.743 (0.266–2.080)	0.572		
% positive cores^a^	0.975 (0.833–1.140)	0.7500		
NCCN unfavourable (vs. favourable)	1.170 (0.634–2.16)	0.616		
Any cribriform pattern^b^	1.43 (0.671–3.040)	0.355		
Small cribriform pattern only^b^ ^,^ ^c^	0.623 (0.204–1.910)	0.408	0.362 (0.1070–1.220)	0.101
Non‐small cribriform pattern^b^ ^,^ ^c^	3.430 (1.210–9.690)	0.0201	2.420 (0.8170–7.140)	0.111
Highest %GP4^a^	1.55 (1.240–1.950)	0.0002	1.610 (1.2600–2.070)	0.0002
Total length of GP4^d^	1.160 (1.010–1.320)	0.0309		
% GS 7 cores^a^	1.190 (0.916–1.560)	0.191		

^a^ Odds ratio for each 10% increase in each factor.

^b^ Odds ratios indicate comparison to cases with no cribriform pattern.

^c^ These features are included in the same categorical variable together with no cribriform pattern.

^d^ Odds ratio for each 1 mm increase in GP4‐TL.

### 3.3 Correlations among the three different quantification methods of Gleason Pattern 4

The Spearman's rank correlation coefficient was 0.738 (*P* < 0.0001) between the highest %GP4 and GP4‐TL, 0.346 (*P* < 0.0001) between the highest %GP4 and percentage of GS 7 cores and 0.570 (*P* < 0.0001) between the GP4‐TL and percentage of GS 7 cores.

### 3.4 Cut‐off value analyses with receiver operating characteristic curve

The cut‐off value for the highest %GP4, which was the sole independent predictive factor for AP, was calculated using the ROC curve. The ‘sensitivity + specificity’ was maximal, and ‘(1 − sensitivity)^2^ + (1 − specificity)^2^’ was minimal when the highest %GP4 was 20% (Figure S1).

## 4 DISCUSSION

In the current study, we evaluated the predictive value of preoperative clinicopathological parameters for AP among cases of GS 3 + 4 = 7 IR‐PCa. Previously, we examined the clinicopathological significance of the highest %GP4 and GP4‐TL in GS 3 + 4 = 7 PCa, including high‐risk cases.^5^, ^21^ In these studies, we divided GS 3 + 4 = 7 cases into subgroups by any cut‐off value of the highest %GP4, GP4‐TL and number of GS 7 cores and compared outcomes between each GS 3 + 4 = 7 subgroup and GS 6 cases. As a result, the frequency of AP was not significantly different between GS 7 cases with %GP4 < 5% and GS 6 cases.^5^ In addition, the frequency of AP was also not significantly different between the highest %GP4 > 5% cases with GP4‐TL < 0.5 mm and GS 6 cases.^21^ However, these studies did not consider other clinicopathological parameters including CP. In this study, we conducted a more detailed and comprehensive analyses in a multivariate manner to show the predictive value of clinicopathological parameters. Further, high‐risk cases were excluded to be more clinically relevant. The current study's findings suggest that the highest %GP4 and GP4‐TL were predictive factors of AP in univariate analysis. Although many studies have shown the predictive value of %GP4, studies focused on GS 3 + 4 = 7 IR‐PCa cases are limited. Cole et al. reported that %GP4 in biopsy specimens was an independent predictive factor of AP in IR‐PCa with GS 3 + 4 = 7,^10^ and Perlis et al. also reported similar results.^8^ These results demonstrate the definite predictive value of %GP4 in IR‐PCa and support our results. Furthermore, research on the clinicopathological significance of GP4‐TL is limited. Dean et al. have reported the predictive value of GP4‐TL, which was an independent predictive factor of AP in cases with GS 3 + 4 = 7 at biopsy.^22^ Although the results of our studies and those described by Dean et al. suggest that GP4‐TL is a potent prognostic factor in GS 3 + 4 = 7 IR‐PCa patients, further verification should be performed before clinical application.

In the current study, multivariate analysis showed that the highest percentage of GP4 was the sole predictive factor for AP. However, GP4‐TL, which was predictive of AP in the univariate analysis, was not predictive in the multivariate analysis. Although GP4‐TL showed a relatively strong correlation with the highest %GP4 with a Spearman's rank correlation coefficient of 0.738, the statistical results outlining the predictive value of GP4‐TL should be reliable since the index was under 0.8. From a practical clinicopathological aspect, the highest %GP4 is simpler and more understandable than GP4‐TL because GP4‐TL requires complicated calculations. Regarding the clinicopathological utility of GP4‐TL, our previous study showed that GP4‐TL is useful only in a limited setting.^21^ Thus, we conclude that the highest %GP4 is a superior predictive factor, considering our current results and practical aspects comprehensively.

Regarding the clinicopathological significance of CP, any/small CP was not predictive of AP, even in univariate analysis. Non‐small CP was predictive of AP in univariate analysis, although this association did not show statistical significance in multivariate analysis. In the setting of GS 3 + 4 = 7 IR‐PCa, a few studies have shown that CP is not a significant predictor of postoperative GS upgrade and/or pT3 disease.^23^, ^24^ In contrast, many studies that included high‐risk/higher‐grade diseases have shown that CP is a worse prognosticator.^7^, ^11^, ^12^, ^23^, ^24^, ^25^ This disparity may be explained by the higher incidence of intraductal carcinoma, which is included in the CP category in most studies, and by CP itself in higher‐grade diseases.^7^ Based on these results, the clinicopathological significance of CP may be rather small in the GS 3 + 4 = 7 IR‐PCa setting. Regarding the size of CP, several previous studies utilising radical prostatectomy specimens of GS 7 have shown that the biological significance was different between small and non‐small CP.^26^, ^27^ Although only one study by Flood et al. showed that small CP in biopsy specimens was associated with adverse outcomes compared with GS 6 cases, they categorised both cases with and without non‐small CP under the small CP category.^17^ Thus, their results do not reflect the genuine biological features of small CP. Our current study and other studies suggest that cases with small CP may show different biological behaviours than those with non‐small CP. Accordingly, different clinical approaches may be recommended for small‐ and non‐small CP cases.

The current study, which examined the predictive value of clinicopathological factors for AP among cases of GS 3 + 4 = 7 IR‐PCa, offers significant evidence that contributes to the timely subclassification of IR‐PCa. This is the first study which shows predictive superiority of %GP4 over the CP and other clinicopathological parameters in cases with GS 3 + 4 = 7 IR‐PCa. However, the study has some limitations, including a single‐institute design and a small cohort size of 228 cases, leading to reduced statistical power. Another limitation was that the pathological outcome of AP was adopted as an outcome measure. Because the cohort included recent cases, clinically significant prognostic outcomes were not adopted. In addition, a lower inter‐observer reproducibility rate for poorly formed glands of GP4 is another challenge to be overcome before clinical application.^28^ Further verification with a larger cohort is required to compare clinical prognostic outcomes.

## 5 CONCLUSION

In the current study, only the highest %GP4 was an independent predictive factor for AP in GS 3 + 4 = 7 IR‐PCa. Thus, the highest %GP4 may be used as a predictive factor in the classification of IR‐PCa.

## DISCLOSURE OF INTEREST

Takahiro Kimura is a paid consultant/advisor of Astellas, Bayer, Janssen and Sanofi. Shin Egawa is a paid consultant/advisor of Takeda, Astellas, AstraZeneca, Sanofi, Janssen and Pfizer. The other authors declare no conflicts of interest associated with this manuscript.

## AUTHOR CONTRIBUTIONS

SS designed and performed study and drafted the entire manuscript, tables and figures. TK supervised the research and revised the draft from a clinical perspective. HO collected the clinical data and supervised statistics. SE supervised the research from a clinical perspective. MS supervised the research from a pathological perspective. HT supervised the research and revised the draft from a pathological perspective.

## Supporting information


**Figure S1.** Receiver operating characteristic curve for the predictive value of adverse pathological findings by the highest percentage of Gleason pattern 4.

**Table S1.** Distribution of highest percentage of Gleason pattern 4 in biopsy specimens
