# Supplementary material for: The highest percentage of Gleason Pattern 4 is a predictor in intermediate‐risk prostate cancer
Source: BJUI Compass. 2022 Oct 17;4(2):234–40. doi: 10.1002/bco2.195 (PMC9931537; doi:10.1002/bco2.195)
Supplement: Supplementary file 1 — Figure S1. Receiver operating characteristic curve for the predictive value of adverse pathological findings by the highest percentage of Gleason pattern 4. [file BCO2-4-234-s002.pptx]

## Slide 1
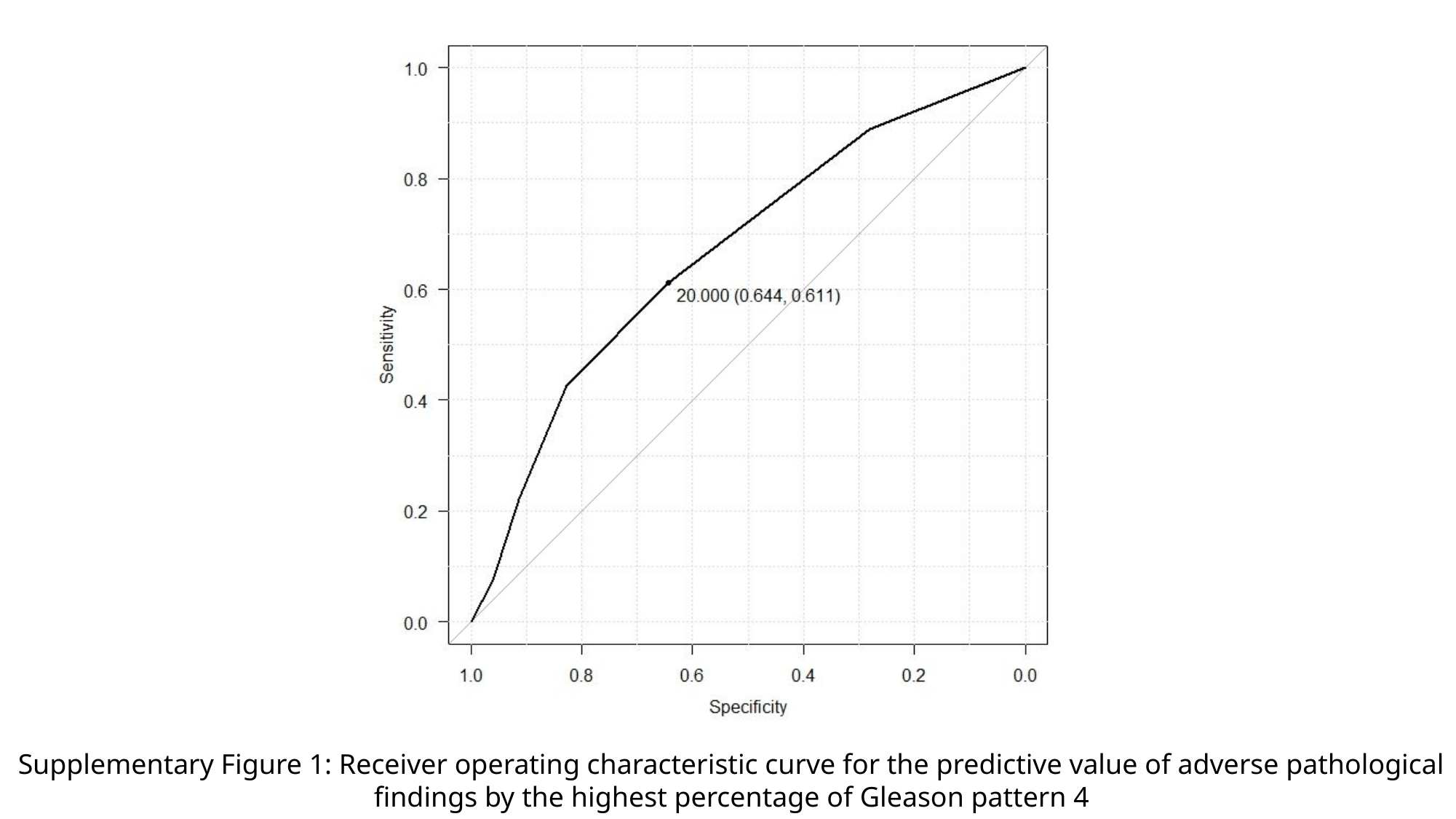

Supplementary Figure 1: Receiver operating characteristic curve for the predictive value of adverse pathological findings by the highest percentage of Gleason pattern 4
