# Supplementary material for: The highest percentage of Gleason Pattern 4 is a predictor in intermediate‐risk prostate cancer
Source: BJUI Compass. 2022 Oct 17;4(2):234–40. doi: 10.1002/bco2.195 (PMC9931537; doi:10.1002/bco2.195)
Supplement: Supplementary file 2 — Table S1. Distribution of highest percentage of Gleason pattern 4 in biopsy specimens [file BCO2-4-234-s001.docx]

| Supplementary table 1. Distribution of highest percentage of Gleason pattern 4 in biopsy specimens | |
| --- | --- |
| Highest percentage of Gleason pattern 4 | Case number (%) |
| <5% | 55 (24.1) |
| 10% (5-15%) | 78 (34.2) |
| 20% (15-25%) | 42 (18.4) |
| 30% (25-35%) | 26 (11.4) |
| 40% (35-45%) | 16 (7.0) |
| ≧45% | 11 (4.8) |
